# Effects of soil resource availability on patterns of plant functional traits across spatial scales

**DOI:** 10.1002/ece3.8587

**Published:** 2022-02-14

**Authors:** Yanpeng Li, Han Xu, Jie Chen, Yihua Xiao, Yunlong Ni, Ruyun Zhang, Wanhui Ye, Juyu Lian

**Affiliations:** ^1^ Key Laboratory of Vegetation Restoration and Management of Degraded Ecosystems Guangdong Provincial Key Laboratory of Applied Botany, South China Botanical Garden Center for Plant Ecology, Core Botanical Gardens Chinese Academy of Sciences Guangzhou China; ^2^ Forest Ecology Research Center Research Institute of Tropical Forestry Chinese Academy of Forestry Guangzhou China; ^3^ Southern Marine Science and Engineering Guangdong Laboratory (Guangzhou) Guangzhou China

**Keywords:** environmental gradient, plant functional trait, soil resource availability, spatial scale, the stress‐dominance hypothesis

## Abstract

Identifying patterns and drivers of plant community assembly has long been a central issue in ecology. Many studies have explored the above questions using a trait‐based approach; however, there are still unknowns around how patterns of plant functional traits vary with environmental gradients. In this study, the responses of individual and multivariate trait dispersions of 134 species to soil resource availability were examined based on correlational analysis and torus‐translation tests across four spatial scales in a subtropical forest, China. Results indicated that different degrees of soil resource availability had different effects on trait dispersions. Specifically, limited resource (available phosphorus) showed negative relationships with trait dispersions, non‐limited resource (available potassium) showed positive relationships with trait dispersions, and saturated resource (available nitrogen) had no effect on trait dispersions. Moreover, compared with the stem (wood density) and architectural trait (maximum height), we found that leaf functional traits can well reflect the response of plants to nutrient gradients. Lastly, the spatial scale only affected the magnitude but not the direction of the correlations between trait dispersions and environmental gradients. Overall, the results highlight the importance of soil resource availability and spatial scale in understanding how plant functional traits respond to environmental gradients.

## Graphical abstract

In this study, the responses of individual and multivariate trait dispersions of 134 species in Dinghushan plot, China, to soil resource availability were examined. Results showed that different degrees of soil resource availability had different effects on trait dispersions. Specifically, limited resource mainly showed negative relationships with trait dispersions, non‐limited resource mainly showed positive relationships with trait dispersions, and saturated resource had no effect on trait dispersions.

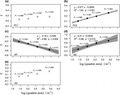

## 1 INTRODUCTION

Identifying the patterns and drivers of plant community assembly has long been a central issue in ecology (Chapman & McEwan, 2018; Chesson, 2000). There is a growing consensus that the assembly of natural plant communities is governed by stochastic (random events related to dispersal, establish, mortality, etc.) and deterministic (Hubbell, 2001; Macarthur & Levins, 1967) processes. In particular, the two deterministic processes environmental filtering and biotic interactions have attracted more attention in community assembly research (Kuczynski & Grenouillet, 2018; Swenson & Enquist, 2009). Environmental filtering increases species similarity through abiotic constraints, while biotic interactions lead to a limitation of the similarity of coexisting species (Gotzenberger et al., 2016; Ramm et al., 2018; Spasojevic & Suding, 2012). Plant functional traits describe ecological differences between different species; however, which plant functional traits could well reflect plant response to environmental gradients and how they respond remain questions (Costa et al., 2017; Wang et al., 2018).

Trait dispersion is a measure of the variation in functional traits within a community. Revealing how trait dispersions vary with environmental gradients is important to advance the predictive ability of functional ecology (Muscarella & Uriarte, 2016). A widely used explanatory proposition for the relationships between trait dispersions and environmental gradients is the stress‐dominance hypothesis (SDH), which predicts that environmental filtering plays a major role in stressful environments, yielding a clustered pattern of trait dispersion, whereas biotic interactions determine community assembly in benign environments, favoring an overdispersed pattern of trait dispersion (Coyle et al., 2014; Swenson & Enquist, 2007; Weiher & Keddy, 1995). Therefore, an increasing trait dispersion pattern from stressful to benign environments should be expected (Costa et al., 2017; Spasojevic & Suding, 2012; Wang et al., 2018). Although many studies have tested the universality of the SDH in forest communities, empirical supports are still contradictory (Coyle et al., 2014; Lhotsky et al., 2016; Spasojevic & Suding, 2012; Wang et al., 2018).

Firstly, testing of the SDH depends on the proxy used to measure environmental gradients (Costa et al., 2017; Lhotsky et al., 2016; Wang et al., 2018). It is noteworthy that different studies often calculate standing biomass as proxies of environmental gradients (Costa et al., 2017; Lhotsky et al., 2016; Liu et al., 2010) and this method implicitly assumes that the environmental stress increases with decreasing standing biomass (Grime, 1977; Gross et al., 2010). However, the variation in standing biomass could be driven by the topography, soil, or microhabitat properties in the research area (Gross et al., 2010; Michalet, 2006), which makes the comparison between different studies without basis and leads to conflicting empirical supports (Li et al., 2019). Secondly, whether the SDH is true for different functional traits is still unknown, because different functional traits may respond differently to environmental gradients (Li et al., 2019; Wang et al., 2018).

Although soil resource availability is a major driving force in community trait structure (John et al., 2007; Katabuchi et al., 2012; Pinho et al., 2018), it has been rarely considered when analyzing how trait dispersions vary with environmental gradients. As the "big three" of crucial soil nutrients, the resource availability of nitrogen (N), phosphorus (P), and potassium (K) strongly affects competition between plant species, as species vary in their ability to cope with different nutrient resources (Koerselman & Meuleman, 1996; Mao et al., 2019). In response to N limitation, legumes could fix atmospheric N in a symbiotic relationship with bacteria (Xu et al., 2019), and plant species could also coexist by varying in the form in which they preferentially absorb N (e.g., NO_3_^−^, NH_4_^+^, or organic N) in an N‐limited community (Ehrenfeld et al., 2005). Moreover, plants can respond in two ways to overcome P limitation: increasing P‐use efficiency aboveground, and/or adjusting their P‐uptake strategies, including root morphological, physiological, and biotic adaptations (Ehrenfeld et al., 2005; Kitayama, 2013; Vitousek et al., 2010). Recent studies have revealed that plants that had the highest mycorrhizal dependency could be supplied 90% P by mycorrhizal fungi (van der Heijden et al., 2006, 2015). Plants could also absorb nutrients over their needs to prevent other species from taking over (Van Wijk et al., 2003). For instance, when there is plenty of K in the soil then plants secure it more than the normal amount and these reserves could be used to support growth when external nutrients are not available (Chapin, 1980). Overall, plants evolve a variety of strategies to cope with the change in soil resource availability, which can ultimately be reflected in the distribution patterns of plant functional traits (Suding et al., 2005).

**FIGURE 1:**
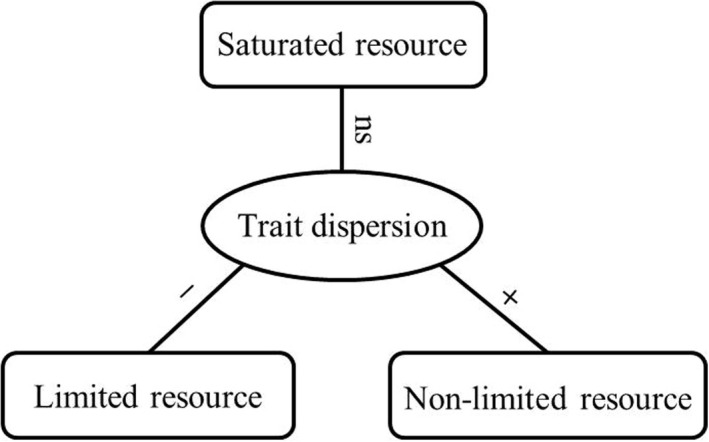
Conceptual models of the effects of soil resource availability on trait dispersion. Limited resource has a significant negative effect on trait dispersion from more stressful to less stressful habitats; Non‐limited resource has a significant positive effect on trait dispersion from less benign to more benign habitats; saturated resource has no significant effect on trait dispersion from less saturated to more saturated habitats

Here, we tested how trait dispersions varied with soil resource availability, which may contribute to reconciling the contrasting relationships between trait dispersions and environmental gradients. We hypothesized that different degrees of soil resource availability (limited resource, non‐limited resource, and saturated resource) should have different effects on trait dispersion patterns. Firstly, trait dispersion should be highest in habitats with the lowest concentration of limited resources, where competition for those resources is strongest (Figure 1). It means that the less the limited resources, the more the intense competition between plants. Empirical studies have shown that plants in infertile soils produce more roots to increase the competitive ability for belowground nutrients (Chen et al., 2021; Schenk, 2006). Secondly, non‐limited resources should have significant positive effects on trait dispersions from less benign to more benign habitats (Figure 1). In fact, plants can adjust energy allocation between shoots and roots in different environments (Campbell et al., 1991). Previous studies found that aboveground competition for light was most intense in benign habitats (Grime, 1977; Weiss et al., 2019; Wilson & Tilman, 1993) and nutrient addition (single nutrient or multiple nutrients in combination) ultimately shifted biotic interactions from belowground competition for nutrients toward aboveground competition for light (Harpole et al., 2016). Lastly, saturated resources should have no significant effects on trait dispersions because these resource supplies exceed the biological demands (Figure 1).

It is undeniable that trait dispersion may depend on which functional traits are involved (Li et al., 2019; Wang et al., 2018). As different functional traits are often related to different ecological strategies (Violle et al., 2007), testing our hypotheses based on single and multivariate trait dispersions may provide more insights about particular niche axes (Lhotsky et al., 2016; Spasojevic & Suding, 2012). Moreover, the detectability of trait dispersion is also scale‐dependent (Cavender‐Bares et al., 2009; Weiher & Keddy, 1995; Zhang et al., 2018). For example, trait overdispersion has been more often detected at small spatial scales where competitive adversity predominates (Li et al., 2019; Price et al., 2017). Additionally, previous studies also found that the relative importance of environmental variables to community assembly increased with increasing spatial scales (Chase, 2014; Legendre et al., 2009). Thus, multiple spatial scale analysis is helpful to evaluate the relationships between trait dispersions and environmental gradients, since a large number of quadrats represent a wide range of spatial variability in soil resource availability and species composition (Coyle et al., 2014).

The 20‐ha subtropical forest plot in Dinghushan (DHS), China, is characterized by the following soil features: (1) limited available phosphorus (AP) due to low soil pH caused by nitrogen deposition and highly weathered soil feature of the old age of this forest, (2) non‐limited available potassium (AK) and (3) saturated available nitrogen (AN) due to long‐term high nitrogen deposition in this region (Fang et al., 2006; Koerselman & Meuleman, 1996; Lin et al., 2013; Lu et al., 2010; Mo et al., 2006). All of these soil features provide an ideal background for examining how trait dispersions vary with soil resource availability. Here, we analyzed trait dispersion patterns of 134 species across six functional traits and four spatial scales while taking into account the edaphic data. We hypothesized that: (1) given that plants respond differently to the change of soil resource availability, different degrees of soil resource availability should have different effects on trait dispersion patterns. Specifically, the limited AP should have negative effects on trait dispersions; the non‐limited AK should have positive effects on trait dispersions; and the saturated AN should have no significant effects on trait dispersions across spatial scales; (2) as different functional traits are often related to different ecological strategies, their responses to environmental gradients may differ markedly; and (3) because both trait dispersion and environmental gradient are scale‐dependent, their relationships may vary with the spatial scale.

## 2 MATERIALS AND METHODS

### 2.1 Study location

The study was conducted in the 20‐ha (500 m × 400 m) plot, which was established between December 2004 and April 2005 in Dinghushan Nature Reserve (23°09′21″–23°11′30″N, 112°30′39″–112°33′41″E), Guangdong Province, China (Li et al., 2019). This forest is well protected from anthropogenic disturbance for over 400 years and is treated as climax vegetation in south China (Zhang et al., 2018). The mean annual temperature in this plot is 20.9°C, with the highest monthly average temperature being 28.0°C in July and the lowest being 12.6°C in January. The mean annual precipitation is 1929 mm, approximately 70% of which falls between April and September (Li et al., 2019). The topography in the DHS plot is very complex with elevations varied from 237.1 m to 466.2 m, convexity varied from −13.4 to 17.7 degree, and slope ranging from 4.4 to 88.6 degree at 20 m × 20 m spatial scale (Zhang et al., 2018). The tree census in this plot followed a standard protocol and included all stems with a diameter at the breast height (*DBH*) larger than 1.0 cm at 1.3 m above the ground (https://www.forestgeo.si.edu/). Every individual tree in the plot has been revisited every 5 years since 2005. Here, the first tree census data collected in 2005 were used in the following analyses with a total number of 71,336 individuals from 51 families, 110 genera, and 183 species.

### 2.2 Functional trait measurement

For the 134 species that accounted for 99.0% of all individuals with DBH ≥ 1.0 cm, six plant functional traits including a stem trait (wood density, WD), an architectural trait (maximum height, Hmax), and four leaf traits: leaf area (LA), specific leaf area (SLA), leaf dry matter content (LDMC), and leaf thickness (LT), were measured (Li et al., 2019; Shen et al., 2016). Traits selected in this study are expected to be good predictors of the response of plant species to variation in resource availability (Li et al., 2019; Pinho et al., 2018; Poorter et al., 2008). LA (cm^2^) is related to light acquisition (Hao et al., 2018). SLA (cm^2^ g^−1^) is a good indicator of the potential relative growth rate of plants (Hao et al., 2018; Wright et al., 2004). LDMC (g g^−1^) is associated with leaf life span and correlated with leaf resistance (Cornelissen et al., 2003; Vaieretti et al., 2007). LT (mm) has important consequences for leaf water content and nutrient cycling (Afzal et al., 2017; Pérez‐Harguindeguy et al., 2016). WD (g cm^−3^) represents a trade‐off between low construction costs and high growth rates vs. high construction costs and low growth rates (Poorter et al., 2008). Hmax (m) is indicative of plant competitive vigor and strategy (Hao et al., 2018). Wood samples for each species followed Cornelissen et al. (2003), and WD was calculated as the ratio of dry mass to fresh volume. Specifically, 6–12 individuals were randomly selected for each species and 10 mature leaves for each individual were measured (Li et al., 2019). Detailed sampling protocols for these six functional traits were described in previous studies (Li et al., 2019; Shen et al., 2016). We calculated the mean value of each trait for each species (Li et al., 2019) to represent the characteristics of species because of the larger variations in functional traits between than within species (Garnier et al., 2001; Shipley, 2007).

### 2.3 Soil data measurements

Because of the thin soil layer in the DHS plot, topsoil (0–10 cm depth) was collected using a 5.0‐cm‐diameter soil auger after removing the litters on the soil surface based on regular grids of 30 m × 30 m (Lin et al., 2013). Each of the 238 grid points was paired with two additional sample points at 2, 5, or 15 m in a random compass direction from the grid to capture fine‐scale variation in soil properties (John et al., 2007). In total, 710 soil samples (four of 714 samples were not taken because they fell in creeks or on rocks) were collected and nine soil properties were measured (Lin et al., 2013), including soil moisture (SM), organic matter (OM), soil pH (pH), total phosphorus (TP), available phosphorus (AP), total potassium (TK), available potassium (AK), total nitrogen (TN), and available nitrogen (AN). Kriging methods were used to obtain the predicted values of the nine soil properties for each quadrat at 5 m × 5 m, 10 m × 10 m, 20 m × 20 m, and 50 m × 50 m spatial scale (Gallardo, 2003). Descriptive statistics of the edaphic variables across four spatial scales are given in Table 1.

**TABLE 1:** Descriptive statistics of the edaphic variables across four spatial scales in the 20‐ha Dinghushan plot, China

	5 m × 5 m	10 m × 10 m	20 m × 20 m	50 m × 50 m
pH	3.75 ± 0.08	3.75 ± 0.08	3.75 ± 0.09	3.74 ± 0.09
SM (%)	18.84 ± 2.67	18.83 ± 2.67	18.80 ± 2.71	18.72 ± 2.59
OM (g kg^−1^)	60.95 ± 10.93	60.97 ± 11.01	60.98 ± 11.21	61.22 ± 11.74
AN (mg kg^−1^)	201.74 ± 35.50	201.74 ± 35.73	201.79 ± 36.26	201.99 ± 37.30
AP (mg kg^−1^)	1.81 ± 1.06	1.81 ± 1.05	1.80 ± 1.04	1.77 ± 1.00
AK (mg kg^−1^)	55.04 ± 19.20	55.00 ± 19.30	54.99 ± 19.58	54.81 ± 19.85
TN (g kg^−1^)	1.18 ± 0.49	1.18 ± 0.50	1.18 ± 0.51	1.19 ± 0.52
TP (g kg^−1^)	0.28 ± 0.05	0.28 ± 0.05	0.28 ± 0.05	0.28 ± 0.06
TK (g kg^−1^)	18.28 ± 3.34	18.24 ± 3.35	18.15 ± 3.41	17.89 ± 3.34

Note

The mean value ± standard deviation of each variable at each spatial scale was shown.

### 2.4 Statistical analysis

#### 2.4.1 Comparing the observed trait dispersions with those from null models

Trait dispersion is quantified by a multidimensional functional diversity index: functional dispersion (FDis) (Laliberté & Legendre, 2010). FDis is defined as the mean distance of individual species to the centroid of all species in the multidimensional trait space within a given community (Laliberté & Legendre, 2010). Among several available metrics of functional diversity (Mouchet et al., 2010), the main reasons why FDis was selected were as follows: Firstly, FDis is independent of species richness (Zhang et al., 2018); furthermore, FDis can take relative abundances of the species into account (Wang et al., 2018); finally, FDis can be used for single or multiple traits (Spasojevic & Suding, 2012). Moreover, FDis well represents the degree of trait dissimilarity among coexisting species, and thus, it is closely related to the strategies of resource utilization (Chiang et al., 2016; Hao et al., 2018).

Before the analysis, all of the six functional traits were rescaled to center on zero with a standard deviation of 1 to eliminate the effects of the magnitudes of the data on the calculation of FDis (Hao et al., 2018; Villeger et al., 2008). Besides, once the species‐species Euclidean distance matrix is obtained from the species–trait matrix, a principal coordinates analysis (PCoA) is then performed on the distance matrix, and the resulting PCoA axes were used as the new “traits” together with a species–abundance matrix to compute the FDis (Hao et al., 2018; Laliberté & Legendre, 2010). Detailed algorithms on how to perform multivariate dispersions in PCoA space from the species–species Euclidean distance matrix and how to correct for negative eigenvalues can refer to Anderson (2006). FDis was calculated by the R package “*FD*” (Laliberté et al., 2014). To test whether any observed trait dispersion is a random distribution or shows trait clustering or trait overdispersion within a quadrat, we generated 999 random assemblages for each quadrat, keeping the same number of species abundances and occurrence frequency in the DHS plot and only randomly shuffling taxon names (Yang et al., 2014). The null models were run by the R package “*picante*” (Kembel et al., 2010). Based on the 999 random assemblages, a standardized effect size of FDis (ZFDis) for each quadrat following Gurevitch et al. (1992) was calculated:

$$\text{ZFDis}=\frac{\left({\text{FDis}}_{\text{observed}}‐{\text{FDis}}_{\text{random}}\right)}{{\text{FDis}}_{\text{sd}}}$$

where FDis_observed_ and FDis_random_ represent the observed FDis and mean FDis values of the simulated 999 random assemblages, respectively. FDis_sd_ represents the standard deviation of FDis values generated from the 999 random assemblages. Positive and negative ZFDis values represent trait overdispersion and trait clustering, suggesting that community assembly is dominated by biotic interactions and environmental filtering, respectively (Swenson, 2014; Wang et al., 2018). Firstly, ZFDis for a multivariate trait that considered all traits in combination was calculated. Secondly, ZFDis for each individual trait was also quantified. It should be noted that the trait dispersions in the DHS plot were calculated at four spatial scales: 5 m × 5 m, 10 m × 10 m, 20 m × 20 m, and 50 m × 50 m. To provide context for our trait‐based analyses, descriptive statistics (e.g., the mean, minimum, and maximum values) for species abundance and richness across spatial scales are given in Table 2.

**TABLE 2:** Mean species abundance and richness across four spatial scales in the 20‐ha Dinghushan plot, China

Spatial scale	Number of quadrats	Abundance (stem)	Richness (species)
5 m × 5 m	8000	9.02 ± 0.06 (1, 45)	5.85 ± 0.03 (1, 24)
10 m × 10 m	2000	35.34 ± 0.38 (1, 114)	14.14 ± 0.11 (1, 33)
20 m × 20 m	500	141.29 ± 2.55 (30, 358)	28.07 ± 0.32 (10, 55)
50 m × 50 m	80	883.08 ± 31.31 (324, 1648)	55.60 ± 1.06 (39, 79)

Note

The values out of the brackets represent mean ± SE, and those in the brackets represent the minimum and maximum values, respectively.

#### 2.4.2 Principal components analysis

Because the axis scores of PCA based on soil factors were usually calculated as indicators of environmental gradients (Costa et al., 2017; Coyle et al., 2014; John et al., 2007), we also conducted the PCA from the R package “*vegan*” (Oksanen et al., 2017) for the nine measured soil variables. To determine how many PCA axes should be retained, Horn's parallel analysis was also conducted and components with adjusted eigenvalues greater than 1 are retained (Dinno, 2018). Finally, the retained PCA axes were selected as one kind of composite indicator of the environmental gradients.

#### 2.4.3 Correlational analysis and torus‐translation test

Pearson's correlation coefficient (*r*) was calculated between trait dispersion and each of the five environmental variables (PC1, PC2, AN, AP, and AK) at four spatial scales. We tested whether *r* between trait dispersion and each environmental variable at each spatial scale was significant using torus‐translation tests, which take into account the inherent spatial autocorrelation in both trait dispersions and environmental variables (Harms et al., 2001). Torus‐translation tests compare observed *r* between trait dispersion and each environmental variable with *r* predicted under a null model in which the trait dispersion is distributed randomly with respect to quadrat. To obtain the predicted values of *r*, each spatial distribution map of an environmental variable is overlaid on the trait dispersion map at each spatial scale, and translated while the trait dispersion map remains fixed, and the edges of the distribution map of each environmental variable wrap back on each of the four cardinal directions (up, down, left, and right) (Comita et al., 2007). With the 20 m × 20 m spatial scale in this plot (consisting of 500 20 m × 20 m quadrats) for example, 500 unique torus‐translated distribution maps of each environmental variable were initially possible (including the true distribution map of each environmental variable). From this, it is possible to generate three original maps to continue this two‐dimensional torus translation: mirror image, 180 rotation, and 180 rotation of the mirror image. In total, these procedures provide another 1500 translated maps (not including the true distribution map of each environmental variable), each of which provides a predicted *r* value. *p*‐values were calculated based on the number of times that the observed *r* was higher or lower than the predicted *r* values (Comita et al., 2007). If the observed *r* value was lower than 5.0% or higher than 95.0% of the expected *r* values in a given spatial scale, then we could infer that the environmental variable has a significant negative or positive effect on trait dispersion at a significance level of 0.05. Overall, a total of 32,000, 8000, and 320 translated maps were generated at 5 m × 5 m, 10 m × 10 m, and 50 m × 50 m spatial scale, respectively. For each environmental variable, *r* values were plotted against the spatial scales (quadrat area), and their trends were tested by fitting linear regression models. All analyses were conducted in R 3.5.1 (R Core Team, 2018).

## 3 RESULTS

### 3.1 Environmental gradients

**FIGURE 2:**
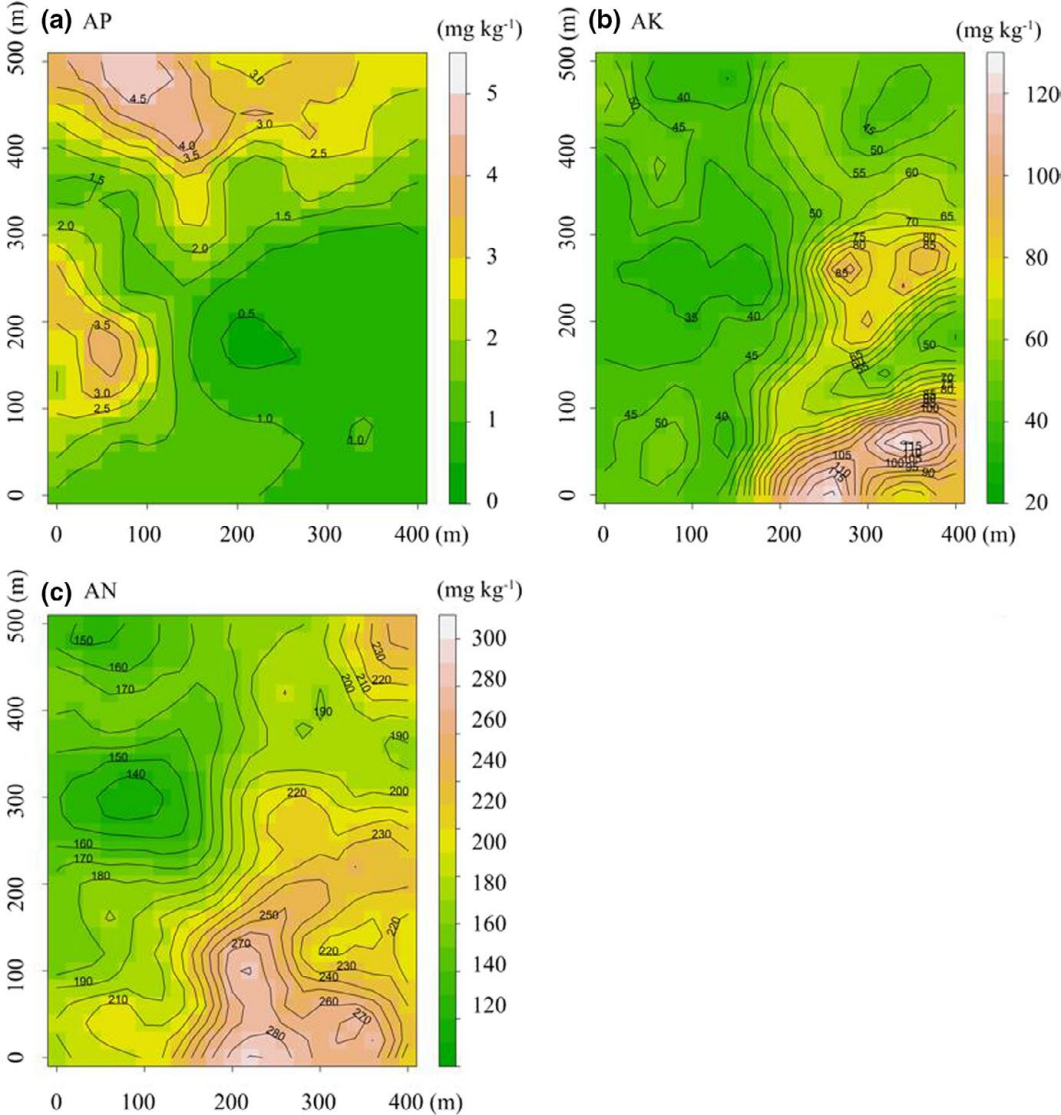
Spatial distribution patterns of AP (a), AK (b), and AN (c) in the 20‐ha DHS plot, China

AP in DHS plots ranged from 0.40 to 4.87 mg kg^−1^ (Figure 2a); AK ranged from 30.11 to 121.18 mg kg^−1^ (Figure 2b); AN ranged from 133.26 to 291.09 mg kg^−1^ (Figure 2c). This set of edaphic factors was also described by the PCA. According to Horn's parallel analysis, 2 axes should be retained for all spatial scales considered except for 50 m × 50 m spatial scale (Figure S1). To ensure the consistency of analysis, the first two axes of PCA for each spatial scale were selected for further analysis. Specifically, they explained 77.9%, 78.0%, 77.9%, and 79.1% of the variance across four spatial scales, respectively (Table 3). In all cases, PC1 showed a soil fertility gradient from infertile to fertile habitats (Table 3). It should be addressed that AP, pH, and SM were negatively correlated with the PC1 (Table 3). PC2 represented a gradient from stressful (low TK and SM) to benign (high TK and SM) conditions (Table 3).

**TABLE 3:** Factor loadings of the first two axes of principal components analysis on edaphic variables across four spatial scales

	5 m × 5 m		10 m × 10 m		20 m × 20 m		50 m × 50 m	
	PC1	PC2	PC1	PC2	PC1	PC2	PC1	PC2
pH	−0.362	0.175	−0.361	0.175	−0.359	0.175	−0.352	0.185
SM (%)	−0.184	0.671	−0.186	0.670	−0.188	0.669	−0.211	0.650
OM (g kg^−1^)	0.371	−0.016	0.371	−0.015	0.372	−0.014	0.373	−0.014
AN (mg kg^−1^)	0.391	0.059	0.391	0.057	0.391	0.055	0.389	0.041
AP (mg kg^−1^)	−0.308	−0.057	−0.307	−0.056	−0.306	−0.053	−0.300	−0.035
AK (mg kg^−1^)	0.343	0.092	0.343	0.092	0.344	0.094	0.346	0.112
TN (g kg^−1^)	0.395	−0.024	0.394	−0.020	0.394	−0.014	0.392	0.014
TP (g kg^−1^)	0.379	0.002	0.379	0.001	0.379	−0.002	0.378	−0.005
TK (g kg^−1^)	0.182	0.709	0.183	0.710	0.183	0.712	0.190	0.726
Cumulative proportion	0.647	0.779	0.648	0.780	0.649	0.779	0.666	0.791

### 3.2 Trait dispersion patterns along with spatial scales

**FIGURE 3:**
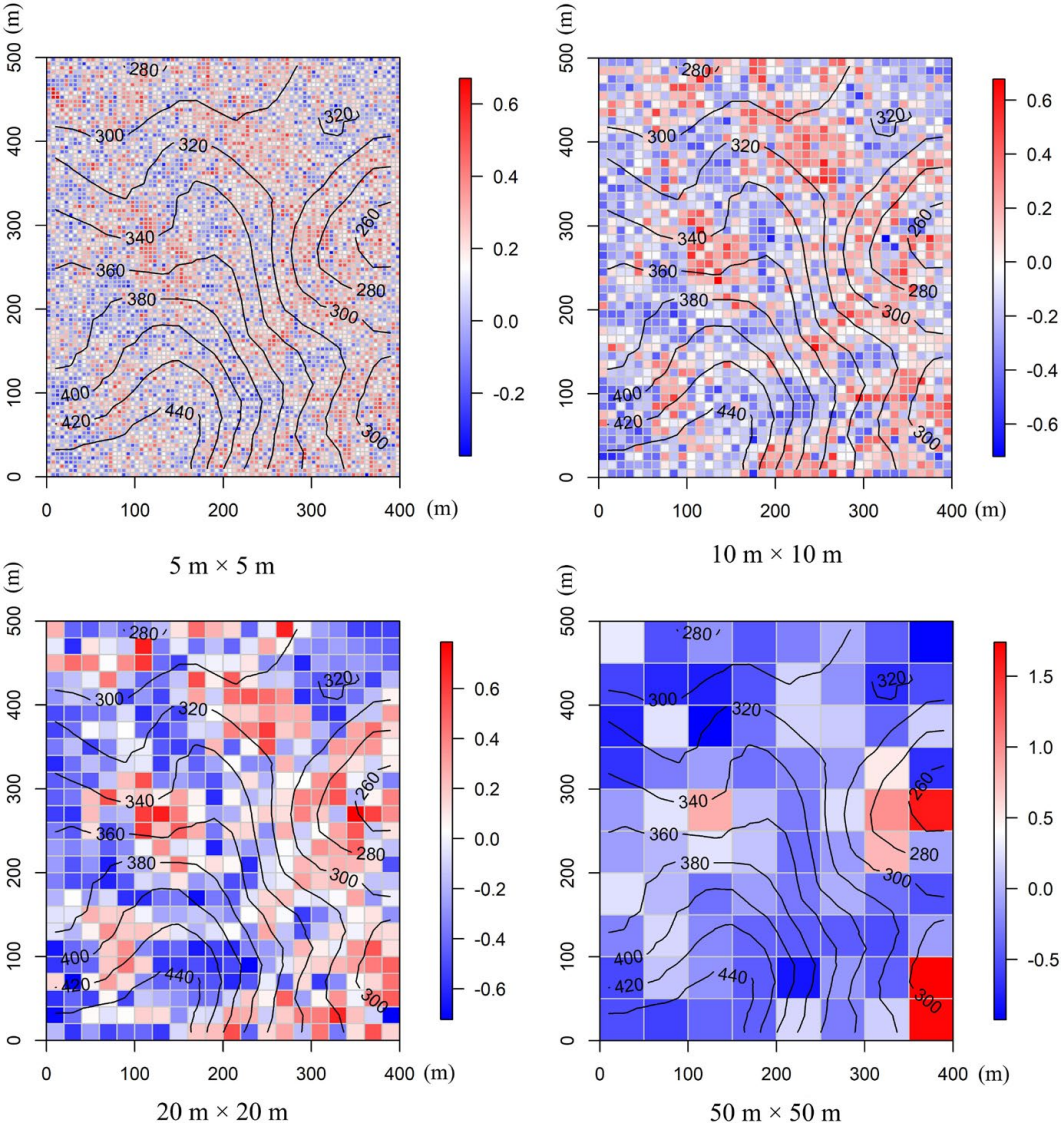
Distribution patterns of the multivariate trait dispersion (ZFDis, the standardized effect size of trait dispersion) at four spatial scales. The color bar on the right of each map indicates the ZFDis values. “ZFDis > 0” indicates that trait dispersion is overdispersed, and “ZFDis < 0” indicates that trait dispersion is clustered. The lines in each map represent the contour lines at 20‐m intervals

We quantified ZFDis for a multivariate trait that considered all traits in combination. Overall, trait dispersions in our plot showed both trait clustering and overdispersion patterns across four spatial scales (Figure 3). Moreover, interquartile ranges of the multivariate trait dispersions were 0.31, 0.39, 0.52, and 0.55 at 5 m × 5 m, 10 m × 10 m, 20 m × 20 m, and 50 m × 50 m spatial scale, respectively. ZFDis for each trait individually was also quantified, and the interquartile ranges of these trait dispersions also increased with increasing spatial scales except for LA (Table 4). Overall, a wider range of spatial variability in patterns of plant functional traits was detected with the increasing spatial scale.

**TABLE 4:** Interquartile ranges of trait dispersions for individual trait across four spatial scales in the 20‐ha Dinghushan plot, China

	5 m × 5 m	10 m × 10 m	20 m × 20 m	50 m × 50 m
Hmax	0.33	0.38	0.55	0.86
LA	0.25	0.24	0.21	0.24
SLA	0.30	0.36	0.46	0.62
LDMC	0.31	0.35	0.42	0.48
LT	0.33	0.34	0.42	0.73
WD	0.29	0.34	0.41	0.81

Abbreviations: Hmax, maximum height; LA, leaf area; LDMC, leaf dry matter content; LT, leaf thickness; SLA, specific leaf area; WD, wood density.

### 3.3 The effects of soil resource availability on trait dispersions

**FIGURE 4:**
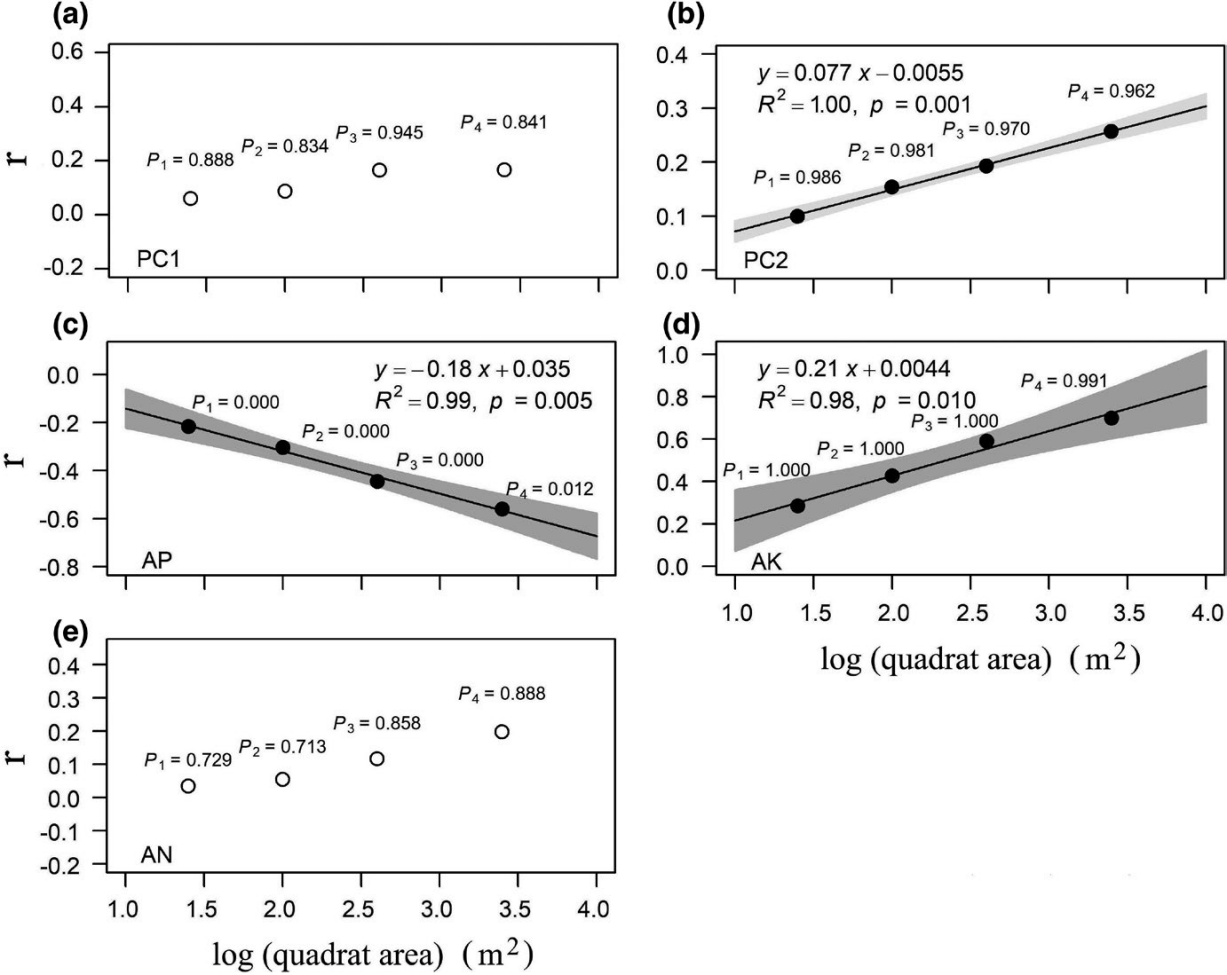
Pearson's correlation coefficient (*r*) between different environmental variables and multivariate trait dispersion (ZFDis, the standardized effect size of trait dispersion) across four spatial scales. Black and white circles, respectively, indicate significant and nonsignificant effects of environmental variables on trait dispersions at a significance level of 0.05 using torus‐translation tests. Lines are trends fitted by the linear regression models, and the shaded areas represent 95% confidence intervals

Different degrees of soil resource availability showed distinct relationships with the multivariate trait dispersions across spatial scales (Figure 4). Specifically, PC2 and non‐limited AK showed significant positive relationships with multivariate trait dispersions (Figure 4b,d) and limited AP had significant negative relationships with trait dispersions across spatial scales (Figure 4c). However, neither PC1 nor saturated AN exhibited significant correlations with trait dispersions across spatial scales (Figure 4a,e).

**FIGURE 5:**
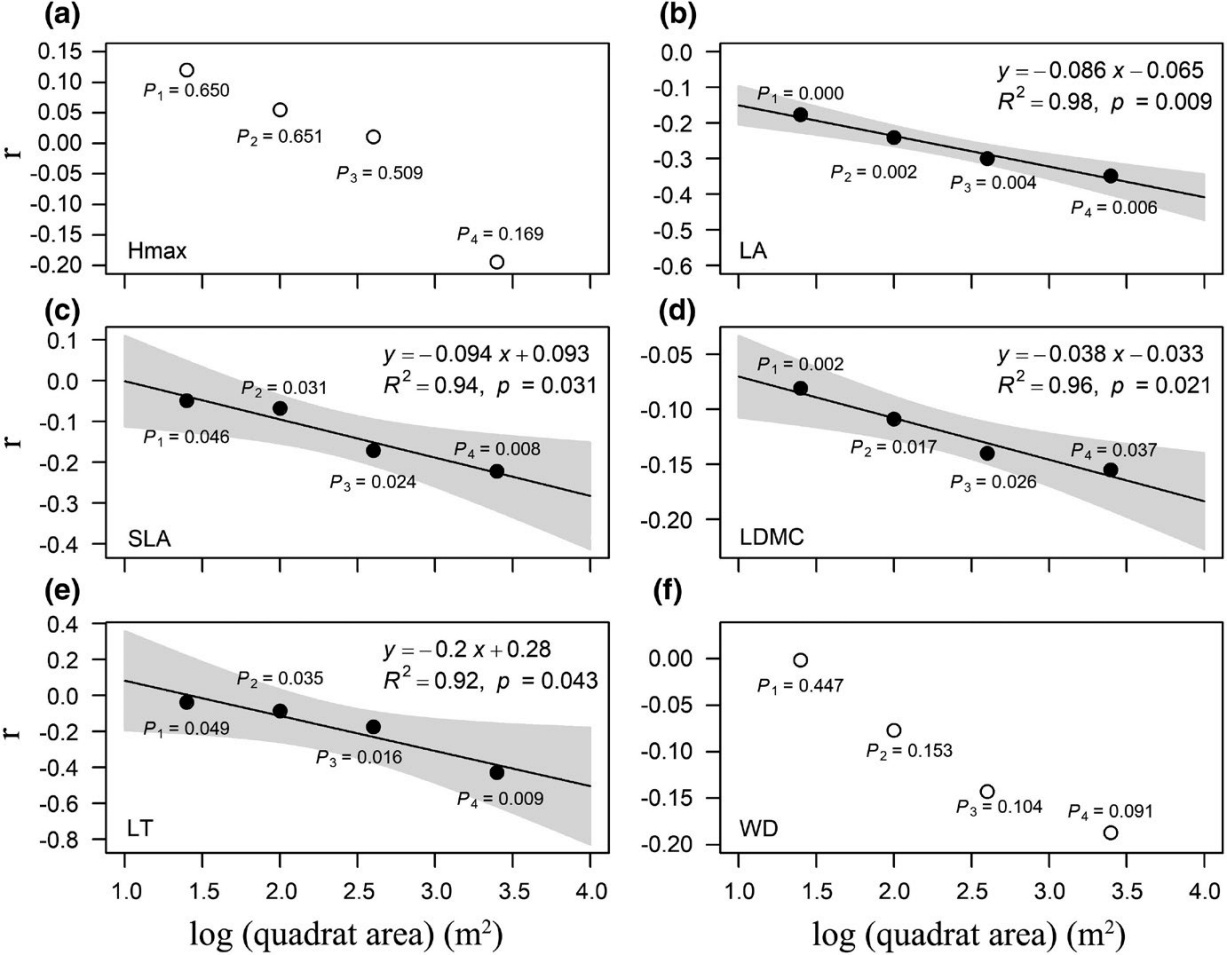
Pearson's correlation coefficient (*r*) between limited AP and individual trait dispersion (ZFDis, the standardized effect size of trait dispersion) across four spatial scales. Black and white circles, respectively, indicate significant and nonsignificant effects of environmental variables on trait dispersions at a significance level of 0.05 using torus‐translation tests. Lines are trends fitted by the linear regression models, and the shaded areas represent 95% confidence intervals

**FIGURE 6:**
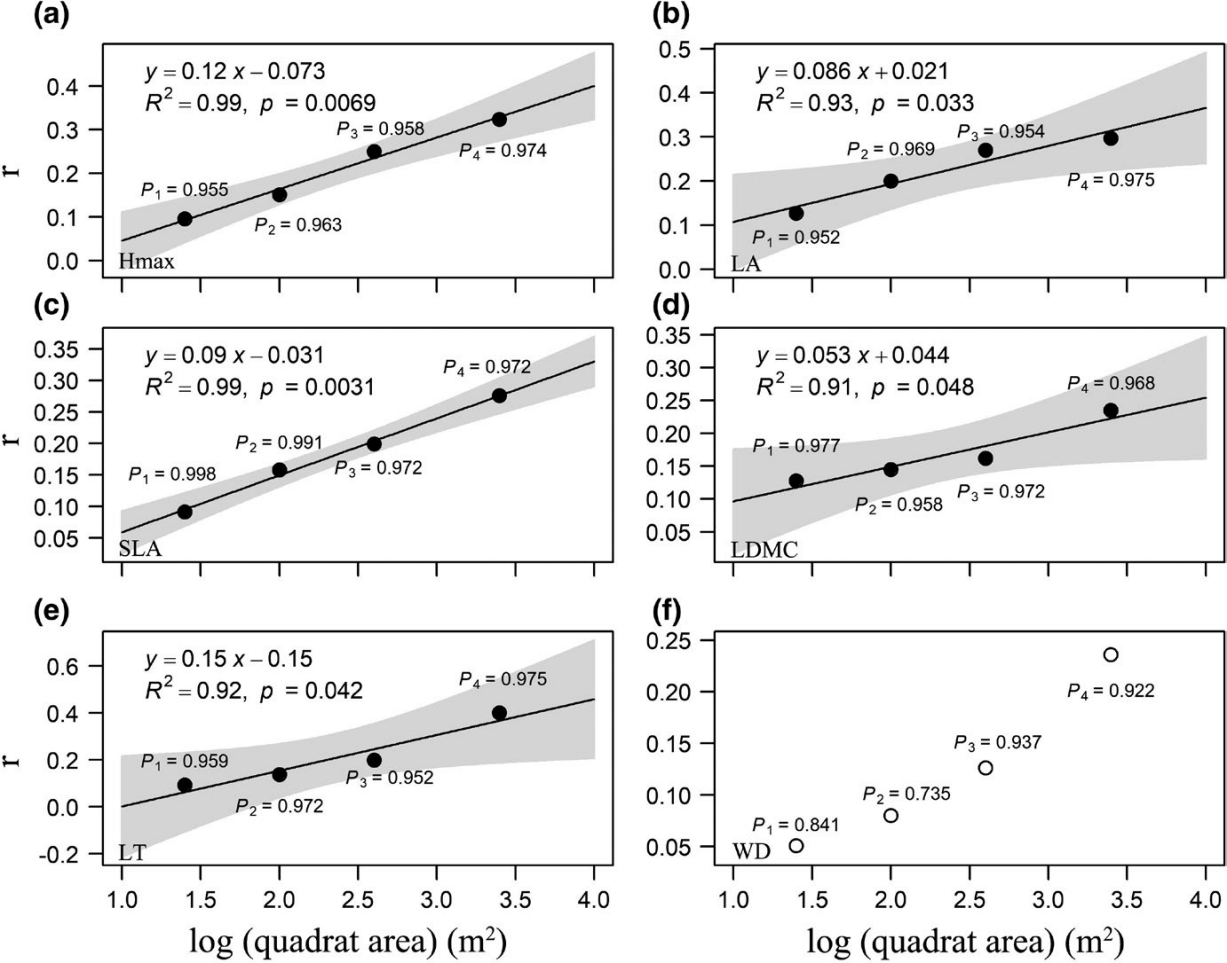
Pearson's correlation coefficient (*r*) between non‐limited AK and individual trait dispersion (ZFDis, the standardized effect size of trait dispersion) across four spatial scales. Black and white circles, respectively, indicate significant and nonsignificant effects of environmental variables on trait dispersions at a significance level of 0.05 using torus‐translation tests. Lines are trends fitted by the linear regression models, and the shaded areas represent 95% confidence intervals

**FIGURE 7:**
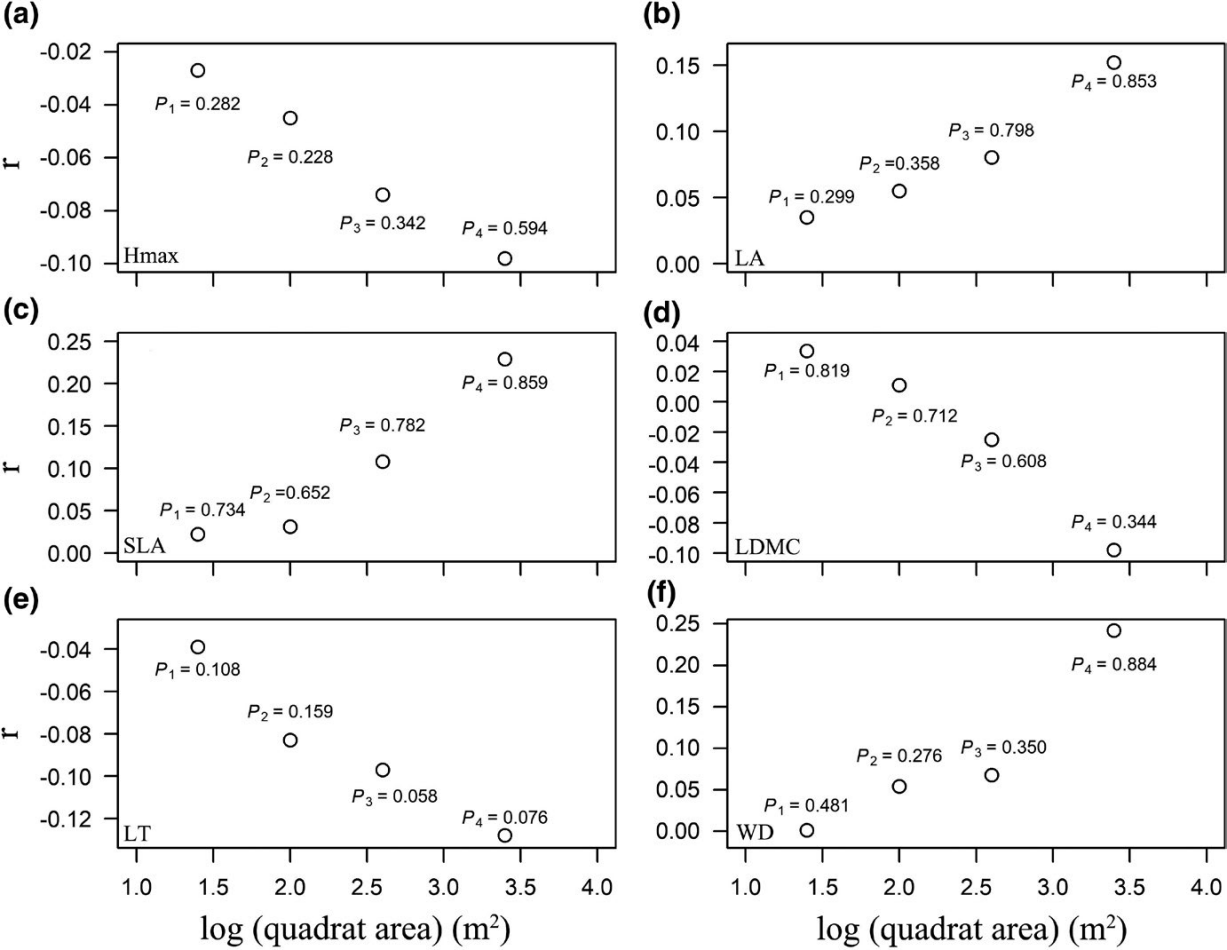
Pearson's correlation coefficient (*r*) between saturated AN and individual trait dispersion (ZFDis, the standardized effect size of trait dispersion) across four spatial scales. Black and white circles, respectively, indicate significant and nonsignificant effects of environmental variables on trait dispersions at a significance level of 0.05 using torus‐translation tests. Lines are trends fitted by the linear regression models, and the shaded areas represent 95% confidence intervals

Moreover, the effects of soil resource availability on most patterns of individual leaf functional traits showed similar results with the multivariate trait dispersion (Figures 5, 6, 7). However, the architectural trait (Hmax) and stem trait (WD) mainly showed no responses to environmental gradients (Figures 5, 6, 7). It should be pointed out that trait dispersions of Hmax showed positive correlations with AK across spatial scales (Figure 6). Overall, the magnitudes of correlation between environmental variables and trait dispersions increased from small to large spatial scales, while directions of correlation between environmental variables and trait dispersions did not change from small to large spatial scales (Figures 5, 6, 7).

## 4 DISCUSSION

Many studies have stressed the importance of abiotic environments to patterns of plant functional traits (Chapman & McEwan, 2018; Liu et al., 2013; Uriarte et al., 2004), but how trait dispersions respond to soil resource availability remains elusive. Our results found that the relationships between trait dispersion and environmental gradient depended on soil resource availability and spatial scale.

### 4.1 The effect of limited soil resource on patterns of plant functional traits

P limitation often occurs in tropical and subtropical forest ecosystems (Mo et al., 2006; Yu et al., 2018). Here, we expected that the less the soil resource availability, the more the intense belowground competition would be (Chen et al., 2021; Tilman, 1982; Zemunik et al., 2015), leading to an increasing trait dispersion pattern. Consistent with our hypothesis, limited soil resource (AP) showed negative relationships with the multivariate trait dispersions across spatial scales in this subtropical forest (Figure 4), indicating the increasing importance of niche partitioning for belowground resources in limited AP habitats. A similar result was also observed in the alpine tundra (Spasojevic & Suding, 2012). Wilson and Tilman (1993) also found that belowground competition was most intense in the lowest N availability plots and decreased significantly with N addition in an N‐limited sand plain.

P is primarily derived from bedrock weathering, and its availability declines with the increase in soil age (Wright et al., 2011; Yu et al., 2018). Foliar N:P ratio has been proposed as an effective indicator of P limitation (Güsewell, 2004; Koerselman & Meuleman, 1996). Liu et al. (2013) reported the mean value of foliar N:P ratios in this forest was 29.6, which far exceeded the critical threshold of 12.5 to 26.3 (Tessier & Raynal, 2003). Moreover, the average content of AP is 1.81 mg kg^−1^ (Figure 2a), indicating severe P limitation in the DHS plot.

Here, we also explored how individual trait responded to P limitation. Compared with the stem (WD) and architectural traits (Hmax), leaf traits are more sensitive to P limitation and these trait dispersions were highest in the most P‐deficient habitats. Except for fulfilling a structural role, P plays an integral role in photosynthesis, plant growth, and resistance to pathogens and abiotic stresses (Kitayama, 2013; Thuynsma et al., 2016). Overall, the results showed that leaf functional traits could well reflect the adaptive strategies of plants to survive in low P habitats (Roy‐Bolduc & Hijri, 2011; Thuynsma et al., 2016).

### 4.2 The effect of non‐limited soil resource on patterns of plant functional traits

Plants can store more than normal amounts of K to support growth, and this phenomenon is called luxury consumption (Chapin, 1980). K is considered seldom limited plant growth in the natural community (Koerselman & Meuleman, 1996). The average content of AK is 55.03 mg kg^−1^ (Figure 2b). The moderate supply of AK (Sun, 2005) indicates K is a non‐limited soil resource in the DHS plot. Thus, we predicted trait dispersions increased with the content of the non‐limited AK from less benign to more benign habitats. In line with our hypothesis, significant positive relationships between AK and the multivariate trait dispersions indicate that the intensity of aboveground competition for light may be greater in more benign habitats (Weiss et al., 2019; Wilson & Tilman, 1993).

The results based on the individual leaf and architectural traits also support our hypothesis. K plays essential roles in plant processes, including regulation of plants’ responses to light and stress resistance (Traenkner et al., 2018; Wang et al., 2013). For the leaf traits, LA and SLA are associated with photosynthesis (Hao et al., 2018; Wright et al., 2004), and LDMC and LT are related to plant resistance to physical hazards (Afzal et al., 2017; Vaieretti et al., 2007). Thus, we can infer that competing for more AK not only improves photosynthesis but also increases resistance to biotic and abiotic stress. Moreover, trait dispersion of Hmax also showed a positive relationship with the increasing content of AK, indicating the important role of Hmax in determining the coexistence of species at different vertical layers (Li et al., 2019).

It should be noted that there is still no clear consensus on which functional traits are more related to which ecological processes (Lhotsky et al., 2016; Yang et al., 2018). Because different functional traits may represent different niche axes (Li et al., 2019; Violle et al., 2007), how these functional traits respond to specific environmental gradients remains to be further explored (Spasojevic & Suding, 2012). Overall, our results confirmed that leaf functional traits can well reflect the response of plants to nutrient gradients, while the stem trait (e.g., WD) was not an effective indicator of plant response to nutrient gradients.

### 4.3 The effect of saturated soil resource on patterns of plant functional traits

Consistent with our hypothesis, we found the saturated AN had no significant effects on the individual and multivariate trait dispersions. The average content of AN is 201.76 mg kg^−1^ in the DHS plot (Figure 2c). Due to the increase in intensified anthropogenic activities, total wet N deposition and total dry N deposition were 34.4 kg N·ha^−1^·y^−1^ and 14.2 kg N·ha^−1^·y^−1^ in 2009–2010 in this plot, respectively (Lu et al., 2018). Fang et al. (2006) also pointed out that the DHS plot can be considered as an N‐saturated ecosystem, resulting in increased leaching of various forms of N.

Although N deposition was thought to play a major role in biodiversity loss due to its effects on soil acidification, aluminum mobility, nutrient base cations, and the ratios of N versus other elements in plant tissue (Gress et al., 2007; Tian et al., 2018), we failed to detect the influence of increasing N availability on trait dispersions across spatial scales. There may be two possible explanations. Firstly, the mature forest in this plot is a regional climax forest type and has been protected for more than 400 years, which has become N‐saturated from both the old age of this forest and chronic high‐level N deposition in this region (Fang et al., 2006; Mo et al., 2006). Presumably, the species in this forest may be adapted to this kind of high‐N conditions and their competition for N might be minimal (Lu et al., 2010). This could be supported by the fact that the N contents in both leaves and roots did not significantly increase with chronic N deposition in this plot (Liu, Zhou, et al., 2010). Secondly, in a field experiment that carried out near this plot, Lu et al. (2010) found that N additions only decreased the abundance of understory ferns, moss, and seedlings, but had no significant effects on shrubs and canopy trees. Besides, the effect of increasing N availability on community structure and composition should be time‐dependent (Güsewell, 2005), and detecting generalizable patterns of trait dispersions at different temporal scales should be rewarding.

### 4.4 The effect of PCA axis on patterns of plant functional traits

A surprising finding was that the PC1 that demonstrated soil fertility gradient from infertile to fertile soils had no significant effects on trait dispersions across spatial scales (Figure 4a). Coyle et al. (2014) also found trait dispersion remained constant to the stress gradient of soil nutrient availability, which was calculated using PCA in eastern North American tree communities. However, these results do not necessarily imply that the edaphic conditions have no effects on trait dispersion patterns. In fact, it could be induced by the opposite loadings on this axis, such as AP, pH, and SM (Table 3). For instance, PC2 that represented a gradient from stressful (low TK and SM) to benign (high TK and SM) conditions showed significant positive relationships with trait dispersions across spatial scales. Thus, it may provide a more accurate assessment of the effect of environmental gradients on trait dispersions from the perspectives of a multivariate PCA and soil resource availability.

### 4.5 The effect of spatial scale on relationships between trait dispersion and soil resource availability

We also found that spatial scale only affected the magnitude but not the direction of the correlations between trait dispersions and environmental gradients. The detectability of trait dispersion is scale‐dependent (Weiher & Keddy, 1995; Zhang et al., 2018). Many studies found that trait dispersions tended to change from an overdispersed to a clustered pattern with the increase in spatial scale (Cavender‐Bares et al., 2009; Li et al., 2019). Moreover, community assembly in natural communities is governed by stochastic and deterministic processes (Hubbell, 2001; Macarthur & Levins, 1967). As spatial scale declines to encompass fewer individuals and less environmental heterogeneity, the relative importance of those stochastic events to the community assembly increases (Chase, 2014), resulting in a weak correlation between environmental gradients and trait dispersions at smaller spatial scales. Previous studies also found that the relative importance of environmental variables to community assembly increased with increasing spatial scales (Chase, 2014; Legendre et al., 2009), leading to a stronger correlation between environmental gradients and trait dispersions at larger spatial scales. Thus, multiple spatial scale analysis is helpful to evaluate the relationship between trait dispersions and environmental gradients.

## 5 CONCLUSIONS

Understanding how plant functional traits change along with environmental gradients becomes increasingly important, especially in the contexts of global climate change and the intensifying human activities, which show great impacts on environmental conditions and species composition of communities. The present study was designed to examine how plant functional traits responded to environmental gradients, and we have found that different degrees of soil resource availability have different effects on trait dispersions. Because different functional traits may represent different niche axes (Li et al., 2019; Violle et al., 2007), we also found that leaf functional traits can well reflect the response of plants to nutrient gradients. Lastly, we point out that spatial scale only affects the magnitude but not the direction of the correlations between trait dispersions and environmental gradients. Overall, these findings are essential for a better understanding of the forces that determine the structure and dynamics of natural communities and to advance the predictive theory of functional ecology.

## CONFLICT OF INTEREST

The authors declare that they have no known competing financial interests or personal relationships that could have appeared to influence the work reported in this paper.

## AUTHOR CONTRIBUTIONS

**Yanpeng Li:** Conceptualization (equal); Data curation (equal); Formal analysis (equal); Methodology (equal); Writing – original draft (lead); Writing – review & editing (equal). **Han Xu:** Formal analysis (equal); Visualization (equal); Writing – original draft (equal). **Jie Chen:** Formal analysis (equal); Visualization (equal); Writing – review & editing (equal). **Yihua Xiao:** Visualization (equal); Writing – review & editing (equal). **Yunlong Ni:** Data curation (equal); Validation (equal); Visualization (equal). **Ruyun Zhang:** Data curation (equal); Validation (equal); Visualization (equal). **Wan‐Hui Ye:** Conceptualization (equal); Data curation (equal); Funding acquisition (equal); Investigation (equal); Project administration (equal); Supervision (equal); Validation (equal). **Ju‐Yu Lian:** Conceptualization (equal); Data curation (equal); Investigation (equal); Project administration (equal); Supervision (equal); Visualization (equal); Writing – original draft (equal).

## Supporting information

Fig S1

## Data Availability

The tree census data for the 20‐ha Dinghushan plot in 2005 are available through the online portal at: http://www.forestgeo.si.edu. The data of plant functional traits are openly available in (Shen et al., 2016) at: https://doi.org/10.1017/S0266467416000262, and (Li et al., 2019) at: https://doi.org/10.3390/f10121055.
