# Supplementary figures and images for: Effects of soil resource availability on patterns of plant functional traits across spatial scales
Source: Ecol Evol. 2022 Feb 14;12(2):e8587. doi: 10.1002/ece3.8587 (PMC8844114; doi:10.1002/ece3.8587)

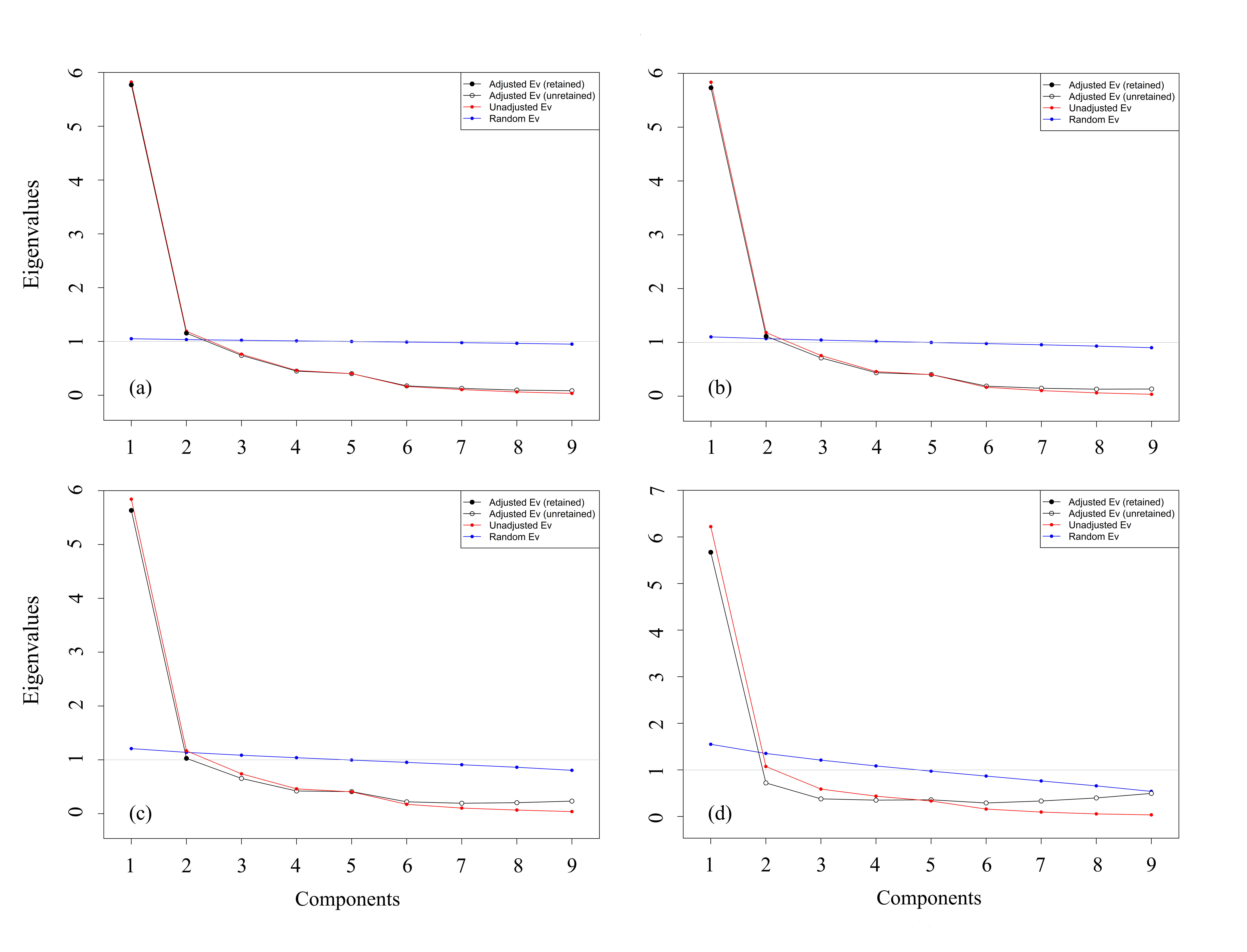

Supplement: Supplementary file 1 — Fig S1 [file ECE3-12-e8587-s001.jpg]
